# Behaviour of *Abutilon theophrasti* in Different Climatic Niches: A New Zealand Case Study

**DOI:** 10.3389/fpls.2022.885779

**Published:** 2022-04-25

**Authors:** Hossein Ghanizadeh, Trevor K. James

**Affiliations:** ^1^School of Agriculture and Environment, Massey University, Palmerston North, New Zealand; ^2^AgResearch, Ruakura Research Centre, Hamilton, New Zealand

**Keywords:** CLIMEX-model, growth degree days, invasive specie, site-specific climate condition, colonisation

## Abstract

*Abutilon theophrasti* Medik. was initially introduced into New Zealand in the 1940s. Despite its introduction approximately 70 years ago, *A. theophrasti* infestation in New Zealand has been naturalized to one region only, although climate-based simulation models predicted that *A. theophrasti* establishment could almost occur in all New Zealand agricultural lands. One possible reason for this discrepancy is that the likelihood of establishment of *A. theophrasti* may vary across various localities as the climate in New Zealand is complex and varies from warm subtropical in the far north to cool temperate climates in the far south. The objective of this research was to assess and compare the likelihood of *A. theophrasti* establishment across various localities in New Zealand. For this, experiments were laid out across different regions in New Zealand to assess vegetative and reproductive characteristics in naturalized and casual populations of *A. theophrasti*. The results showed that the growth and development of both populations varied across different regions, possibly due to variable climatic and geographical conditions such as local temperatures and daily solar radiation. It appears that *A. theophrasti* is, however, able to grow in many regions in New Zealand, but this species is unlikely to establish and become problematic in the lower half of South Island, where the temperature is lower than optimal temperatures required by this species. A casual population was found to grow better at the early stage of growth compared to a naturalized one. However, both populations reproduced similar amounts of seed in all regions. Overall, the variable vegetative and reproductive responses recorded for *A. theophrasti* in different locations may suggest that the invasion dynamic of this weed species is unlikely to be similar across different climatic niches in New Zealand.

## Introduction

The introduction of alien species can threaten the sustainability of local ecosystems. Alien species are either introduced intentionally (e.g., garden ornaments) or accidentally (e.g., imports of contaminated seeds) (Williams and West, 2000). Once introduced, an alien species will colonize and adapt to the local environmental conditions before it establishes and spreads to other areas (Theoharides and Dukes, 2007). Factors such as high growth rate, ability to reproduce over a wide range of environmental conditions, and high dispersal ability facilitate the establishment of an alien species (Clark et al., 2001; Muthukrishnan et al., 2018). However, often, there is a lag phase between local establishment and further spread (Marsico et al., 2010). This lag phase can be due to 1- unfavourable climate conditions of local habitats (Pyšek and Hulme, 2005) 2- the inability of the alien species to rapidly adapt to the local climate owing to a lack of genetic variation (Theoharides and Dukes, 2007) 3- the alien species require time to be overcome genetic constraints such as genetic elements associated with inbreeding depression (Sakai et al., 2001). The lag phase may also reflect the time required by alien species populations to reach the size that allows them to colonize additional locations (Barney, 2006).

In New Zealand, imports of contaminated seeds are one of the major pathways to introduced alien plant species, though the rate of contamination is low due to the strict biosecurity regulations (Rubenstein et al., 2021). However, in some cases, alien plants were intentionally introduced into New Zealand (Hulme, 2020). *Abutilon theophrasti* Medik. is one of the alien plant species that is believed to be introduced into New Zealand in the 1940s as a fibre crop (James and Pene, 2018). *Abutilon theophrasti* is believed to have originated from China, where it was cultivated as a fibre crop (Neal, 1984), but it is currently listed as a noxious weed in many countries across the world (Warwick and Black, 1988). In New Zealand, *A. theophrasti* has also been accidentally imported as seed contaminants with soybean and other grains (James and Cooper, 2012).

*Abutilon theophrasti*, a member of the Malvaceae family, is an annual broad-leaved weed species (Warwick and Black, 1988). It is a troublesome weed species in spring-summer crops such as maize, soybean and cotton (Smith et al., 1990; Lindquist et al., 1998). The growth characteristics and canopy structure of *A. theophrasti* make it a strong competitor for light against agronomic crops (Bazzaz et al., 1989). In addition, prolific seed production and long seedbank persistence of *A. theophrasti* make it a challenging weed species to manage (Lindquist et al., 1996). *Abutilon theophrasti* can cause significant losses in growth and yield when competing with crops. In maize, for example, *A. theophrasti* reduced the yield up to 80% depending on the environment where it occurs (Lindquist et al., 1996). In soybean, *A. theophrasti* resulted in a 66% seed yield loss (Eaton et al., 1976).

*Abutilon theophrasti* exhibits several specific characteristics, including high levels of genetic variability (Warwick and Black, 1988), enhanced competitive ability (Lindquist et al., 1998), allelopathy (Colton and Einhellig, 1980), high fecundity (Lindquist et al., 1995) and high phenotypic plasticity (Wang and Zhou, 2021) that can facilitate its establishment and spread as an alien weed species to new niches (Theoharides and Dukes, 2007). However, despite its introduction approximately 70 years ago, *A. theophrasti* infestation in New Zealand has been restricted to the Waikato and North of Auckland regions in New Zealand (James and Cooper, 2012). Nevertheless, climate-based simulation models predicted that *A. theophrasti* infestation and establishment can almost occur in all New Zealand agricultural lands (Bourdôt and Lamoureaux, 2021). One possible reason for this discrepancy is that the likelihood of establishment of *A. theophrasti* may vary across various localities as the climate in New Zealand is complex and varies from warm subtropical in the far north to cool temperate climates in the far south. To address this, we conducted this research to 1- evaluate the morphological characteristics and growth habit of *A. theophrasti* in different regions in New Zealand and 2- understand potential of *A. theophrasti* to establish across different geographic regions in New Zealand. The spread of *A. theophrasti* can threaten the sustainability of the local ecosystem by competing with resident species, thus evaluating the potential spread of *A. theophrasti* in various local ecosystems obtains a glimpse of hotspots for this species in New Zealand.

## Materials and Methods

### Locations and Plant Material

In this research, the growth and development of two different populations of *A. theophrasti* in different regions in New Zealand were evaluated. Two locations in the North Island (Ruakura and Palmerston North) along with three locations in the South Island (Lincoln, Invermay, and Woodlands) were chosen for this research. One population was chosen from the Waikato region, where *A. theophrasti* has been naturalized (hereinafter referred to as naturalized population) for many years. Another population was chosen from a fodder beet (*Beta vulgaris* L.) site where *A. theophrasti* was recently introduced as a contaminant with fodder beet seeds (hereinafter referred to as casual population) (James and Pene, 2018). We included this casual population as it may carry additional biological and genetic characteristics that enhance its potential establishment compared with the naturalized population. Seeds of the naturalized population were collected from a maize paddock where *A. theophrasti* had been established at least 5 years before seeds were collected. Seeds of the casual population were provided by Ministry for Primary Industries (MPI) and originated from a fodder beet paddock. Both populations were further multiplied up in the glasshouse to obtain enough seeds for this research. Glasshouse grown seeds were collected from mature plants of each population and stored at 4°C with a relative humidity of 35%.

### Phenotypic Traits

Experiments were conducted under natural conditions in five different research centers across New Zealand (Table 1), in September 2018 (Experiment 1) and August 2019 (Experiment 2), representing early spring and late winter emergence, respectively. Monthly total precipitation and average monthly temperatures at the experimental sites during this research are presented in Tables 2, 3, respectively. Average monthly maximum and minimum temperatures at the experimental sites are also presented in Supplementary Table 2. In both experiments, 40 seeds of *A. theophrasti* were sown in a 35-L plastic container (Pöppelmann TEKU, MCD 45, Germany) containing potting media [60% bark, 20% fiber, and 20% Pumice (7 mm)] and slow-release fertilizer (1 kgM^–3^) (Daltons, New Zealand). The fertilizer contained nitrogen (12.0%), phosphate (3.5%), potassium (13.0%), magnesium (11.0%), boron (0.2%), copper (1.0%), iron (15%), manganese (2.4%), molybdenum (0.04%), and zinc (1.0%). There were five pots (five replicates) for each population, and the pots were arranged in a completely randomized design. Supplementary water was provided twice a week during the first 3 months. The pattern of emergence in each pot was monitored and recorded until the end of the experiment. Five to ten days after initial emergence, seedlings were thinned to one seedling per pot and allowed to grow until maturity. There were five pot-replicates to assess phenotypic traits for each population in each experiment. Data were collected on the total thermal time taken to initial emergence, total percentage of emerged seedling, total thermal time taken to reach a height of 100 mm, total thermal time taken to reach a height of 200 mm, total thermal time taken to reach a height of 300 mm, total thermal time taken to initiate flowering, height at flowering, final height, number of branches and number of seed capsules (Supplementary Tables 3, 4). All traits were assessed for every individual plant.

**TABLE 1:** Research facilities in which the experiments were conducted.

Research facility	Coordinates (latitude, longitude)
Ruakura Research Centre- AgResearch	37°46′27.8″S 175°18′31.0″E
Plant Growth Unit- Palmerston North	40°22′37.5″S 175°36′54.4″E
Lincoln Research Centre- AgResearch	43°38′33.1″S 172°28′14.9″E
Invermay Agricultural Centre- AgResearch	45°51′32.1″S 170°23′15.8″E
Woodlands Research Farm- AgResearch	46°21′32.5″S 168°34′55.8″E

**TABLE 2:** Monthly total precipitation at the experimental sites.

	Total rain (mm)				
	Ruakura	Palmerston North	Lincoln	Invermay	Woodlands
September-2018	53	65.6	39.4	50.2	33
October-2018	53.4	53.4	59	55.4	77.8
November-2018	78.6	84.2	104	155.2	124.2
December-2018	184.4	172.6	57	56.8	41.2
January-2019	22.8	29.4	36.2	83.6	48.2
February-2019	14.4	28.8	29.2	19.4	31.6
March-2019	35.4	52.2	26.8	20.2	59.4
August-2019	133	117	41.6	50	4.8
September-2019	151	60.6	36.2	38.6	10
October-2019	66	97.6	49.8	121	48.4
November-2019	57.6	78.8	57.6	52.8	42.6
December-2019	74.8	124.2	37.6	115.6	121.8
January-2020	6.4	31.6	6.6	33.8	17.2
February-2020	9.6	34.8	17.8	110	68

**TABLE 3:** Average monthly temperatures at the experimental sites.

	°C				
	Ruakura	Palmerston North	Lincoln	Invermay	Woodlands
September-2018	23.1	18.6	16.9	16.2	14.7
October-2018	23.0	21.5	19.8	19.3	19.1
November-2018	26.4	25.3	22.5	20.7	19.9
December-2018	30.7	29.5	25.7	24.4	26.4
January-2019	33.8	31.8	30.6	27.6	30.8
February-2019	33.9	30.6	29.7	26.3	27.9
March-2019	31.8	30.3	27.9	26.9	27.2
August-2019	17.8	16.0	14.5	13.8	10.9
September-2019	20.0	18.4	17.2	15.8	14.2
October-2019	22.2	20.5	18.7	17.2	16.4
November-2019	27.5	25.6	25.9	24.0	24.5
December-2019	29.7	27.6	26.0	21.1	22.9
January-2020	30.6	28.8	28.3	24.6	29.9
February-2020	33.9	31.7	29.2	25.4	29.3

### Statistical Analyses

Heterogeneity of variances of data was evaluated using Levene’s test. Seedling emergence and phenological development for plants in different regions were quantified using growing degree days accumulated beginning at emergence (GDD) using the following equation:


$$GDD=\left(\frac{T_{max}+T_{min}}{2}\right)-T_{b}$$

Where *T*_*max*_ and *T*_*min*_ represent maximum and minimum air temperatures recorded on day t, and *T*_*b*_ (base temperature) represent the base temperature for *A. theophrasti* germination. Our preliminary investigation showed that *A. theophrasti* can germinate at temperatures below 5°C (Supplementary Figure 1). Similarly, Loddo et al. (2013) noted that *A. theophrasti* can germinate at a temperature of 4°C. Hence, the base temperature used for *A. theophrasti* in this research was 4°C. The phenotypic data were then subjected to analysis of variance using the univariate procedure (general linear model) of SPSS (IBM SPSS Statistics 27). The total variability for each trait was quantified by using the following model:


$$Y_{ij}=\mu +\alpha_{i}+\beta_{j}+{\left(\alpha \beta \right)}_{ij}+\varepsilon_{ij}$$

where *Y*_*ij*_ is the observation, μ is the mean, α_*i*_ is the effect of population, β_*j*_ is the effect of location, and ε_*ij*_ is the variation due to random error. Means were compared using a least significant difference (LSD) test at *P* = 0.05.

To quantify the relationships between phenotypic traits, a principal components analysis (PCA) was carried out based on all measured traits except for emergence, as it was not correlated with the other variables. Components with eigenvectors > 1 were chosen, and variables with factor loading values > 0.32 were considered to contribute high scores to the component. A permutational multivariate analysis of variance (PERMANOVA) was used to assess if there is a location effect on population traits. The effect of dispersion (within-group variability) was assessed using analysis of homogeneity of multivariate dispersion (PERMDISP). The PERMDISP was performed to check the homogeneity of multivariate dispersion between locations (Anderson, 2006). The PERMANOVA and PERMDISP were conducted using the package *vegan* in R.

## Results

### Emergence Pattern

**FIGURE 1:**
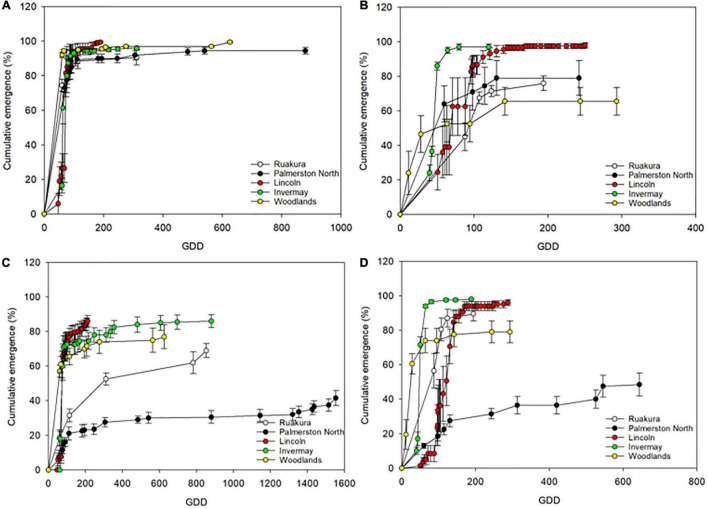
Cumulative seeding emergence of casual **(A,B)** and naturalized **(C,D)** populations of *A. theophrasti* in the first **(A,C)** and second **(B,D)** experiments as related to cumulative growing-degree days (GDD). The experiments were conducted in five different regions of New Zealand. The error bars represent ± standard error of the mean.

The cumulative emergence of naturalized and casual populations of *A. theophrasti* in different locations is illustrated in Figure 1. According to the results, more emerged seedlings were recorded for the casual population than that of the naturalized population in all locations in the first experiment (Figures 1A,C). Also, a similar pattern of seedling emergence (>90%) was recorded across all locations for the casual population in the first experiment. In contrast, the pattern of seedling emergences was varied across different locations for the naturalized population (Figure 1C). In the first experiment, the least number of seedlings emergence for the naturalized population was recorded in Palmerston North, with only 40% emergence recorded by the end of the experiment. In the second experiment, different patterns of seedling emergence were recorded for both populations compared to the first experiment. In the second experiment, over 90% emergence was recorded for the casual population in Invermay and Lincoln. In contrast, the number of emerged seeding in other locations was less than 80%, with Woodlands recruiting the least number of seedlings compared to other locations (Figure 1B). The pattern of seedling recruitment for the naturalized population in all locations in the second experiment was better than that recorded in the first experiment. In the second experiment, over 75% emergence was recorded for the naturalized population in all locations except for Palmerston North where only 50% of seedling emergence was achieved by the end of the experiment (Figure 1D).

In both experiments, the onset of seedling emergence occurred by 50 growing degree days (GDD) for both populations across all locations; however, the GDD accumulation and seedling recruitment pattern differed between both populations (Figures 1A–D). In the first experiment, over 90% of seedling emergence was achieved in all locations by 200 GDD for the casual population (Figure 1A). In contrast, the percentage of seedling emergence for the naturalized population in all locations never reached 90% emergence by the end of the first experiment (Figure 1C). In the first experiment, the cumulative GDD at which emergence ended for the casual population in Lincoln occurred earlier (190 GDD) compared to other locations (Figure 1A). The GDD at which emergence ended for the casual population in the first experiment in Ruakura and Invermay was similar (310 vs. 312 GDDs for Ruakura and Invermay, respectively), while emergence ending occurred at a GDD of 625 and 880, in Woodlands and Palmerston North, respectively (Figure 1A). The cumulative GDD at which emergence ended in the first experiment for the naturalized was, however, different from that of the casual population across all locations (Figure 1B). For the naturalized population, emergence ended at a GDD of 211 in Lincoln, while the cumulative thermal time at which emergence ended in Woodlands, Ruakura and Invermay was 625, 853, and 879 GDDs, respectively. However, in Palmerston North, emergence ending was recorded at a cumulative GDD of 1553 (Figure 1B).

In the second experiment, the emergence ending occurred earlier in Invermay than in the other locations for both populations. The emergence ended at a cumulative GDD of 120 and 188 for the casual and naturalized populations, respectively (Figures 1B,D). The cumulative thermal time at which emergence ended in Ruakura was 194 GDD for both populations in the second experiment. Emergence ending in Palmerston North, Lincoln and Woodlands occurred at 242, 250, and 292 GDDs, respectively, for the casual population in the second experiment. For the naturalized population, the cumulative thermal time at which emergence ended in Lincoln and Invermay was 288 and 293 GDDs, respectively. Whereas In Palmerston North, seedling emergence ended at a greater number of cumulative thermal time (643 GDD). Overall, according to these results, it appears that emergence ending for the casual population achieved at fewer cumulative thermal times compared with the naturalized population, though seedling emergence of both populations required a greater accumulation of heat in Palmerston North and Woodlands compared with other locations.

### Growth Period

**FIGURE 2:**
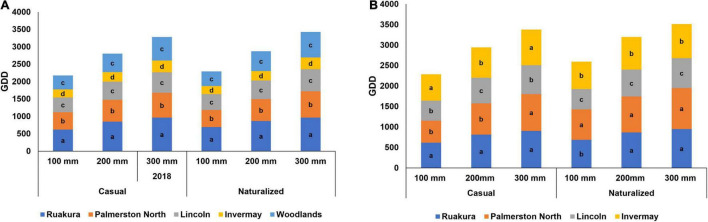
Accumulated growing degree days (GDD) required by the casual and naturalized populations of *A. theophrasti* in five different regions of New Zealand to reach heights of 100, 200, and 300 mm in the first **(A)** and second **(B)** experiments. In the second experiment, plants in Woodlands died after emergence. Within each graph, different letters denote significant (*P* < 0.05) differences between locations.

The early growth stage traits of naturalized vs. casual populations were assessed using the cumulative GDD required to reach 100, 200, and 300 mm for each location (Figure 2). In the first experiment, there were significant differences between populations in the number of cumulative GDD required by plants to reach heights of 100, 200, and 300 mm (*P* < 0.001). Also, the effect of locations on the number of cumulative GDD was significant (*P* < 0.001). However, there were no interactive effects of populations versus locations (*P* > 0.05). For both populations, a slightly greater number of cumulative GDDs was required by plants to reach heights of 100, 200, and 300 mm in Ruakura and Palmerston North compared with other locations (Figure 2A). The least cumulative thermal time to reach heights of 100, 200, and 300 mm was recorded in Invermay, followed by Lincoln and Woodland for both populations in the first experiment.

In the second experiment, a significant difference was recorded for the effect of populations and location on the number of cumulative GDDs required by plants to reach 100, 200, and 300 mm (*P* < 0.001). In addition, the location versus population interaction for cumulative GDD was significant (*P* < 0.001). Both populations required greater cumulative GDDs to reach 100, 200, and 300 mm in the second experiment (Figure 2B). In the second experiment, *A. theophrasti* in Woodland died right after emergence; hence this location was excluded from the analysis. Similar to the results from the first experiments, significantly greater numbers of cumulative thermal times were required by *A. theophrasti* in the North Island (i.e., Ruakura and Palmerston North) to reach 100, 200, and 300 mm, with plants in Ruakura requiring the greatest cumulative thermal time compared with those in other locations (Figure 2B). However, plants in the South Island required lower GDDs (i.e., Lincoln and Invermay) to reach 100, 200, and 300 mm (Figure 2B), with plants in Lincoln requiring the smallest number of cumulative GDDs to establish at the early stage of growth.

**FIGURE 3:**
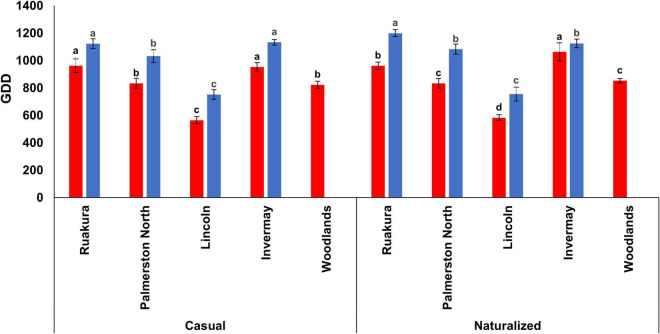
Accumulated growing degree days (GDD) required by the casual and naturalized populations of *A. theophrasti* in five different regions of New Zealand to initiate flowering in the first (red bars) and second (blue bars) experiments. In the second experiment, plants in Woodlands died after emergence. For each population, vertical bars of the same colour with different letters are significantly different at 5% probability. The error bars represent ± standard error of the mean.

In the first experiment, the effects of populations and locations on the cumulative GDDs required for flowering were significant (*P* < 0.001), with the smallest number of cumulative GDDs for flowering recorded in Lincoln for both populations. The results also demonstrated significant interactive effects of populations versus locations (*P* < 0.002) on the number of cumulative GDDs required for flowering. For the casual population, no significant differences were recorded in the number of cumulative GDDs required for flowering between Ruakura (961 GDD) and Invermay (953 GDD). This result implies that greater cumulative GDDs were required for the casual plants to initiate flowering in Ruakura and Invermay compared with the other location (Figure 3). Similarly, the naturalized population in Ruakura and Invermay required greater numbers of thermal time to initiate flowering, though the number of cumulative GDDs in Invermay (1063 GDD) was significantly greater than that of Ruakura (961 GDD). The results from the first experiment also showed that for both populations, there was no significant difference between Palmerston North and Woodlands in the number of GDDs required for flowering (Figure 3).

In the second experiment, the effects of populations and locations on cumulative GDDs required for flowering were significant (*P* < 0.05), with the smallest number of cumulative GDDs required for flowering recorded in Lincoln for both populations. The results, however, demonstrated that there were no significant interactive effects of populations versus locations (*P* = 0.115) on the required cumulative GDDs to initiate flowering. For both populations, the greatest numbers of thermal time required for flowering were recorded for plants in Ruakura and Invermay, though there was a significant difference between both locations in the cumulative GDD for the naturalized population (Figure 3). However, in Palmerston North, both casual and naturalized populations flowered at a similar number of cumulative GDDs in the second experiment.

### Plant Height and Branching

**FIGURE 4:**
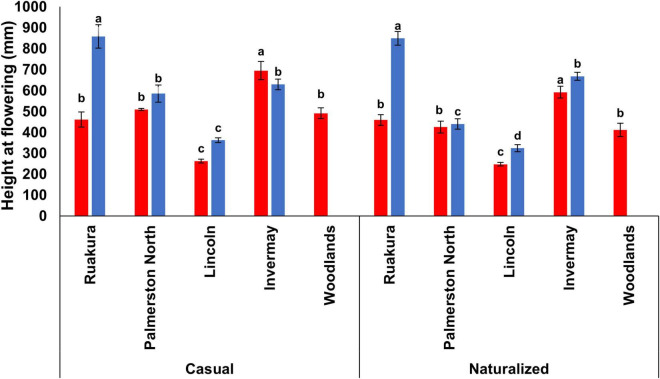
Plant height at flowering for the casual and naturalized populations of *A. theophrasti* in five different regions of New Zealand in the first (red bars) and second (blue bars) experiments. In the second experiment, plants in Woodlands died after emergence. For each population, vertical bars of the same colour with different letters are significantly different at 5% probability. The error bars represent ± standard error of the mean.

In the first experiment, the effects of populations and locations were significant for plant height at flowering (*P* < 0.01). However, the interaction between populations and locations was not significant (*P* = 0.321). For both populations, the tallest and shortest plants at flowering were recorded in Invermay and Lincoln, respectively, in the first experiment (Figure 4). However, the height of plants at flowering was not significantly different between other locations in the first experiment. In the second experiment, only the effect of locations on plant height at flowering was found to be significant (*P* < 0.01). In contrast, the effect of populations on plant height at flowering was not significant (*P* = 0.141). In addition, the interaction between populations and locations was not significant (*P* = 0.118).

**FIGURE 5:**
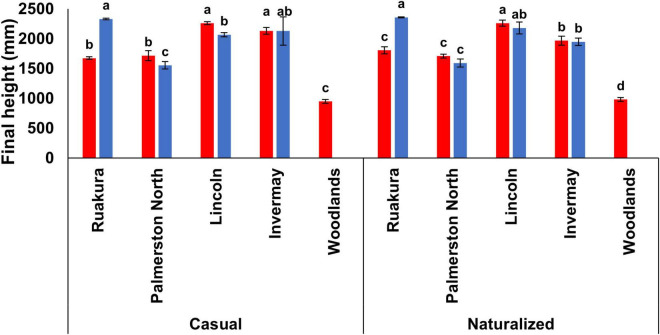
Final plant height for the casual and naturalized populations of *A. theophrasti* in five different regions of New Zealand in the first (red bars) and second (blue bars) experiments. In the second experiment, plants in Woodlands died after emergence. For each population, vertical bars of the same colour with different letters are significantly different at 5% probability. The error bars represent ± standard error of the mean.

The effect of locations on final plant height was significant for both populations in both experiments (*P* < 0.001). In the first experiment, plants were significantly taller in Lincoln (2,260 mm) and Invermay (2,130 mm) compared with other locations (Figure 5) for the casual population. For the naturalized population, plants were significantly taller in Lincoln (2,260 mm) followed by Invermay (1967.5 mm) (Figure 5). For both populations, plants were significantly shorter in Woodlands compared with other locations (Figure 5). However, no significant differences in final plant height were found between Palmerston North and Ruakura for both populations in the first experiment. In the second experiment, the tallest and shortest plants were recorded in Ruakura and Palmerston North, respectively, for both populations (Figure 5). However, the final plant height of plants was not significantly different between Invermay and Lincoln for both populations in the second experiment.

**FIGURE 6:**
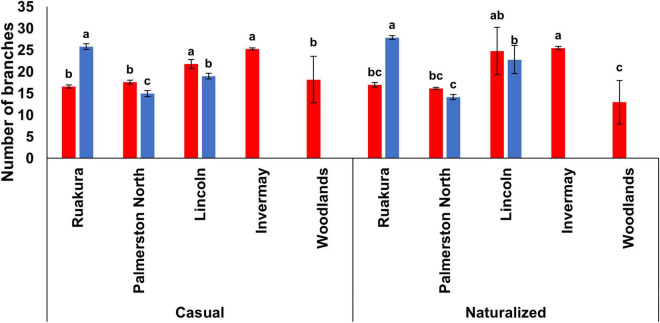
The number of branches for the casual and naturalized populations of *A. theophrasti* in five different regions of New Zealand in the first (red bars) and second (blue bars) experiments. In the second experiment, plants in Woodlands died after emergence. For each population, vertical bars of the same colour with different letters are significantly different at 5% probability. The error bars represent ± standard error of the mean.

In both experiments, the effect of locations on the number of branches was significant for both populations (*P* < 0.01). For the casual population, the highest number of branches was recorded in Lincoln and Invermay in the first experiment, which was significantly different from other locations (Figure 6). For the naturalized population, the number of branches was significantly different between various locations, with the highest and lowest number of branches produced in Invermay and Woodlands, respectively, in the first experiment. In the second experiment, the highest number of branches was produced in Ruakura followed by Lincoln and Palmerston North for both populations (Figure 6). At the same time, no branches were produced in Invermay for both populations in the second experiment.

### Seed Capsule Production

**FIGURE 7:**
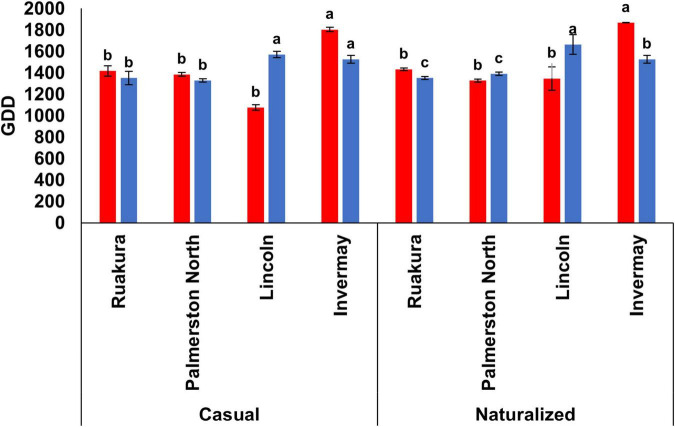
Accumulated growing degree days (GDD) required by the casual and naturalized populations of *A. theophrasti* in five different regions of New Zealand to develop first seed capsules in the first (red bars) and second (blue bars) experiments. No seed capsules were developed by plants in Woodlands in both experiments (in the second experiment, plants in Woodlands died after emergence). For each population, vertical bars of the same colour with different letters are significantly different at 5% probability. The error bars represent ± standard error of the mean.

In the current study, only the effect of locations on the required thermal time for initiating seed capsules was significant in both experiments (*P* < 0.01). In both years, no seed capsules were produced by the plants of both populations in Woodlands. In the first year, *A. theophrasti* in Invermay required a significantly greater heat accumulation for first seed capsule development than other locations (Figure 7). In the second year, plants in both Lincoln and Invermay significantly required more GDDs to develop the first seed capsules compared with Palmerston North and Ruakura (Figure 7).

**FIGURE 8:**
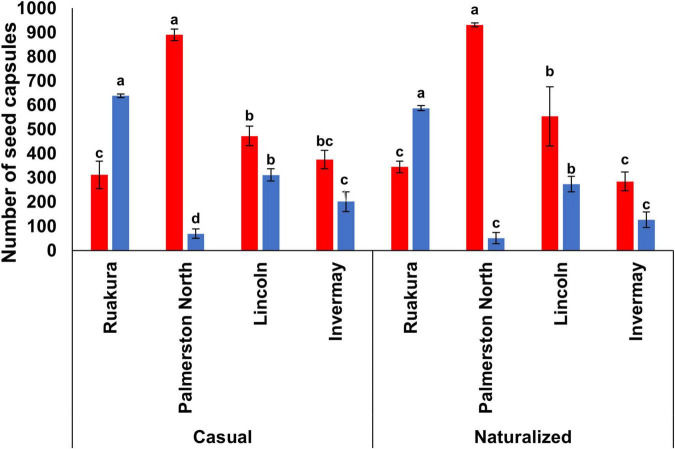
The number of seed capsules for the casual and naturalized populations of *A. theophrasti* in five different regions of New Zealand in the first (red bars) and second (blue bars) experiments. No seed capsules were developed by plants in Woodlands in both experiments (in the second experiment, plants in Woodlands died after emergence). For each population, vertical bars of the same colour with different letters are significantly different at 5% probability. The error bars represent ± standard error of the mean.

In both experiments, the effect of locations on the number of seed capsules was significant for both populations (*P* < 0.01). A significantly higher number of seed capsules was produced in Palmerston North for the casual (890) and naturalized (931) populations (Figure 8) in the first experiment. The second-highest number of seed capsules was recorded in Lincoln, and this was significantly different from Ruakura and Invermay. However, the number of seed capsules was not significantly different between Ruakura and Invermay in the first experiment. In the second experiment, the highest number of seed capsules was significantly produced in Ruakura for both populations (Figure 8). While significantly lower numbers of seed capsules were produced in other locations for both populations, with plants in Palmerston North significantly produced the lowest number of seed capsules.

### Principal Components Analysis

**FIGURE 9:**
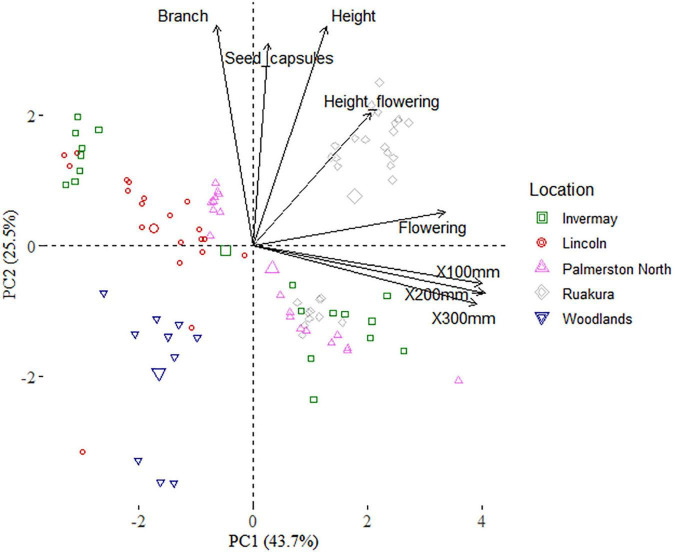
Biplot based on the first two dimensions of the principal component analysis (PCA) of the casual and naturalized populations of *A. theophrasti* in five different regions of New Zealand. Branch = number of branches, Seed_capsules = number of seed capsules, Height = plant final height, Flowering height = plant height at flowering, Flowering = thermal time required to initiate flowering, X100-300 mm = thermal time required to reach a plant height of 100–300 mm.

The first two principal component (PC) axes of the PCA analysis accounted for 69.1% of the variance, with the first component (PC1) explaining 43.7% of the variance (Figure 9 and Table 4). The PC1 was positively associated with the thermal time required to reach 100, 200 and 300 mm, and initiate flowering, indicating that time of flowering initiation is positively correlated with the early-stage growth traits. The principal component 2 (PC2) was positively associated with the number of branches, height at flowering, final height, and the number of seed capsules, reflecting the relationship between seed production and canopy architecture.

**TABLE 4:** Principal component analysis of naturalized and casual populations of *A. theophrasti*.

Variables	PC1	PC2
	
	Loading of traits	
100 mm	0.495	−0.094
200 mm	0.500	−0.117
300 mm	0.483	−0.145
No. of branches	−0.079	0.544
Flowering	0.416	0.081
Final height	0.158	0.542
Height at flowering	0.255	0.329
No. of capsules	0.031	0.500
	
	**Explained variance**	
	
Eigenvalue	1.87	1.43
% of variance	43.7	25.4
Cumulative variance	43.7	69.1

*Loading of characters on the first two axes and explained variance. 100 mm = total thermal time taken to reach 100 mm, 200 mm = total thermal time taken to reach 200 mm, 300 mm = total thermal time taken to reach 300 mm, flowering = total thermal time taken to initiate flowering.*

The biplot illustrates the distribution of the naturalized and casual populations across different locations in the first two principal components (Figure 9). According to the biplot, there is a strong overlap among Palmerston North, Lincoln, and Invermay. There are also some overlaps among Ruakura, Palmerston North, and Invermay. These results indicate that the climate in Woodlands was generally the most different from those of the other locations. PERMANOVA of traits indicated that trait compositions between naturalized and casual populations were dissimilar (*F*(4,93) = 37.8; *R*^2^ = 0.61; *P* = 0.001), and there is an effect of location on traits according to the PERMDISP test (*F*(4,93) = 1.9; *P* = 0.121).

## Discussion

Possessing traits that assist with pre-adaptation to local conditions plays a pivotal role in the establishment of an alien plant species in a new environment (Pyšek and Richardson, 2007). Growth-related traits such as seedling establishment and survivorship over a wide range of environmental conditions, high growth rate, reproductive and dispersal capabilities, and plant height can all contribute to the success of an alien species to establish in a new environment. In this research, we assessed the behaviour of *A. theophrasti* throughout New Zealand. For this, we compared several growth traits between naturalized and casual populations of *A. theophrasti*. The casual population was included because we hypothesized it might have possessed genetic characteristics that enhance its establishment compared to the naturalized population.

The base temperature is the threshold temperature below which no growth and development occur (Steinmaus et al., 2000). Previous studies used a base temperature of 10°C (Lindquist et al., 1998) or 6°C (Tremmel and Patterson, 1994) for *A. theophrasti* to estimate the thermal requirement for growth and development in this species. Loddo et al. (2013) reported that seed germination of *A. theophrasti* can occur at a temperature of 4°C, which agrees with the results of this research. For instance, in Woodlands, in the first experiment, the first emergence flush of *A. theophrasti* was recorded in early October when the average minimum temperature was 3.8°C (Supplementary Table 1). In addition, the results of our preliminary trials revealed that *A. theophrasti* could germinate at 5°C if other conditions, such as ample amounts of water, are met (Supplementary Figure 1). Overall, these results suggest that using a base temperature of 4°C in this research was appropriate.

### The Pattern of Seedling Emergence in Different Locations

This research recorded a greater seedling emergence rate for the casual population in all regions compared with the naturalized population. However, the seedling emergence and establishment pattern was different across all regions for both populations. As the first step of establishment, seedling emergence characteristics play a crucial role in the success of an alien species to occupy a new habitat (Pyšek and Richardson, 2007). It has been suggested that the invasiveness of a species is positively correlated with its germination and emergence rates (Colautti et al., 2006). In agreement with this suggestion, the establishment of alien plant species has been attributed to rapid and profuse seedling emergence in several studies (Forcella et al., 1986; Lambrinos, 2002; Van Kleunen and Johnson, 2007). The better seedling emergence performance recorded for the casual population of *A. theophrasti* in this research may suggest greater potential for this population to invade wider regions in New Zealand compared to the naturalized population.

### The Growth of *Abutilon theophrasti* in Different Locations

In the current research, it was noted that the thermal requirement for the early stage of growth tended to be higher for *A. theophrasti* in the North Island compared with those in the South Island. In addition, *A. theophrasti* seedlings in Woodlands (lower half of South Island) immediately died after emergence when they emerged in late winter. Temperature is a crucial factor for the development of plants (Hatfield and Prueger, 2015). *Abutilon theophrasti* can grow in a wide range of soil types (Warwick and Black, 1988); however, the temperature is a limiting factor for its growth, with cool temperatures (e.g., 17/3°C) inhibiting its growth and development (Flint et al., 1983). According to Table 3, in both experiments, it appears that plants in the South Island regions (i.e., Lincoln, Invermay and Woodlands) experienced lower temperatures at the early stage of growth compared with those in the North Island regions (i.e., Palmerston North and Ruakura). Hence, it is plausible to assume that lower temperatures significantly reduced the growth period for *A. theophrasti* in the South Island. Similar results have been reported for other weed species such as *Amaranthus palmeri* S. Wats. (Spaunhorst et al., 2018), *A. rudis* Saur (Wu and Owen, 2014), and *A. retroflexus* L. (Khan et al., 2021).

The results showed that the time to onset flowering varied depending on regions. The least thermal time required for plants to initiate flowering was recorded in Lincoln for both casual and naturalized populations. Flowering time can be influenced by climatic factors such as minimum and maximum temperatures and geographical factors such as latitude and altitude (Rathcke and Lacey, 1985; Fenner, 1998; Templ et al., 2017). However, it was difficult to relate the same factor to flowering initiation across all regions. For instance, it was noted that the thermal requirement for initiating flowering in Ruakura, and Invermay was greater than in other regions. Yet, the two regions do not share the same climatic and geographical traits. Previously, it was noted that increasing growth temperatures resulted in a delay in the development of *A. theophrasti*, with plants growing at warmer temperatures tending to accumulate greater thermal times before flowering initiation (Patterson, 1992). Presumably, the greater thermal accumulation recorded for the plants in Ruakura is related to temperatures. Based on the average monthly temperature, *A. theophrasti* experienced the warmest growth period in Ruakura compared to the other regions in both years. However, since plants in Invermay experienced cooler temperatures than those in Ruakura, the greater GDD accumulation recorded before flowering time in Invermay is likely related to other factors than temperature.

This research also showed that the canopy architectural responses (height and branching) of *A. theophrasti* varied significantly across different regions. The canopy architecture of *A. theophrasti* is a function of light availability (Bello et al., 1995). Prior studies have shown that shading promotes height but reduces branching in *A. theophrasti* (Rice and Bazzaz, 1989; Benvenuti et al., 1994; Steinmaus and Norris, 2002). According to Supplementary Figure 2, varied daily solar radiation patterns were recorded for different regions during this research. This variation in solar radiation may explain the variation in plant height recorded at different regions. For instance, in the first experiment, *A. theophrasti* in Invermay received less solar radiation than other locations before flowering initiation (∼80 days after flowering). Hence, the Invermay plants were significantly taller than those in other regions. Such highly plastic responses to light availability in *A. theophrasti* can promote the invasive success of this species in environments with heterogeneous light supply.

### Seed Capsule Production of *Abutilon theophrasti* in Different Locations

Successful and prolific seed production is crucial for alien species to establish, survive for more than one season, and spread to new niches (Pyšek and Richardson, 2007). This research showed that the likelihood and quantity of seed production of *A. theophrasti* differed across different regions. Variation recorded in seed capsule production of *A. theophrasti* in different regions can be attributed to differences in environmental conditions among different locations. In this research, temperature and daily solar radiation as potential drivers of variation in seed development (Sato et al., 1995; Templ et al., 2017) varied across all regions. Suboptimal temperatures and low solar radiation can negatively impact seed development by minimizing the photosynthesis capacity of plants (Johnson, 2016). According to Templ et al. (2017), the pattern of minimum (min) and maximum (max) temperatures are good explanatory variables for variation in flowering and reproductive traits. Patterson (1992) showed that *A. theophrasti* failed to produce seed capsules when it was grown under cool temperature regimes (12/4°C and 19/11°C). Similarly, we did not record any seed development for plants in Woodlands. It was noted that the average min temperature in Woodlands was the lowest in both years compared with other locations during the study (Supplementary Table 1), and this may explain the failure in seed production by *A. theophrasti* in this area. In the first year, plants in Palmerston North produced more seeds than those in Ruakura, even though there is no big difference between the two locations in average monthly max/min temperatures. Sato et al. (1995) noted that solar radiation during the growing season plays a crucial role in the seed production of *A. theophrasti*, with plants experiencing fewer less solar radiation during the growing season producing a small number of capsules. This may explain the significantly fewer seeds produced by *A. theophrasti* in Ruakura since they received fewer daily solar radiation compared with those in Palmerston North in the first year (Supplementary Figure 2). In the second year, however, *A. theophrasti* in Ruakura experienced more daily solar radiation compared with those in Palmerston North and as a result, plants in Ruakura yielded significantly more seeds.

Traits associated with the early-stage phase of the establishment, such as seedling emergence rates and rapid growth at the early stage, play a crucial role in the adaptive potential of alien species in a habitat (Pyšek and Richardson, 2007). Species with rapid growth at the early stage are more capable of capturing resources than slow-growing ones (Mangla et al., 2011). The results of this study showed that the casual population of *A. theophrasti* performed better than the naturalized one at the early stage of growth. This suggests that the casual population may establish quicker than naturalized. However, further investigation, especially under field conditions is needed to assess the adaptive potential of the casual *A. theophrasti* in different habitats.

## Conclusion

Previously, based on a climate niche model, it was suggested that *A. theophrasti* could spread throughout New Zealand (Bourdôt and Lamoureaux, 2021). However, predicting the potential distribution of a species based on climatic-based models may not match with the performance of the species in the local sites since the climatic-based models do not account for other determinants of plant invasion such as site-specific conditions and abiotic factors (Paattison and Mack, 2008). For instance, Bourdôt and Lamoureaux (2021) predicted that the lower half of the South Island in New Zealand is climatically suitable for the establishment and invasion of *A. theophrasti*. However, according to the results of this study, *A. theophrasti* is unlikely to become problematic in the lower half of the South Island. The results showed that although the emergence rate was high for *A. theophrasti* in the lower half of the South Island, but the plants failed to produce capsules in both years successfully. This result suggests that it is unlikely for *A. theophrasti* to establish as an alien species in this area. In other locations, the results of this study showed that *A. theophrasti* can potentially establish, which agrees with the model developed by Bourdôt and Lamoureaux (2021). However, the variable vegetative and reproductive responses recorded for *A. theophrasti* in different locations may suggest that the invasion dynamic of this weed species is unlikely to be similar across various regions in New Zealand. However, detailed information is necessary to determine the establishment rate of *A. theophrasti* across larger scales in New Zealand. Future studies that involve field trials across different regions could provide this information.

## Data Availability Statement

The raw data supporting the conclusions of this article will be made available by the authors, without undue reservation.

## Author Contributions

TJ conceived the ideas, designed the methodology, and edited the drafts. HG collected the data, analysed the data, and wrote the first draft of the manuscript. Both authors gave final approval for publication.

## Conflict of Interest

The authors declare that the research was conducted in the absence of any commercial or financial relationships that could be construed as a potential conflict of interest.

## Publisher’s Note

All claims expressed in this article are solely those of the authors and do not necessarily represent those of their affiliated organizations, or those of the publisher, the editors and the reviewers. Any product that may be evaluated in this article, or claim that may be made by its manufacturer, is not guaranteed or endorsed by the publisher.

## Supplementary Material

Supplementary Figure 1 Percent germination of *Abutilon theophrasti* seed at temperatures of 5–40°C.

Supplementary Figure 2 The pattern of daily solar radiation (MJ m^–2^) at the experimental sites in the first **(A)** and second **(B)** experiments.
