# Supplementary figures and images for: Behaviour of Abutilon theophrasti in Different Climatic Niches: A New Zealand Case Study
Source: Front Plant Sci. 2022 Apr 25;13:885779. doi: 10.3389/fpls.2022.885779 (PMC9083271; doi:10.3389/fpls.2022.885779)

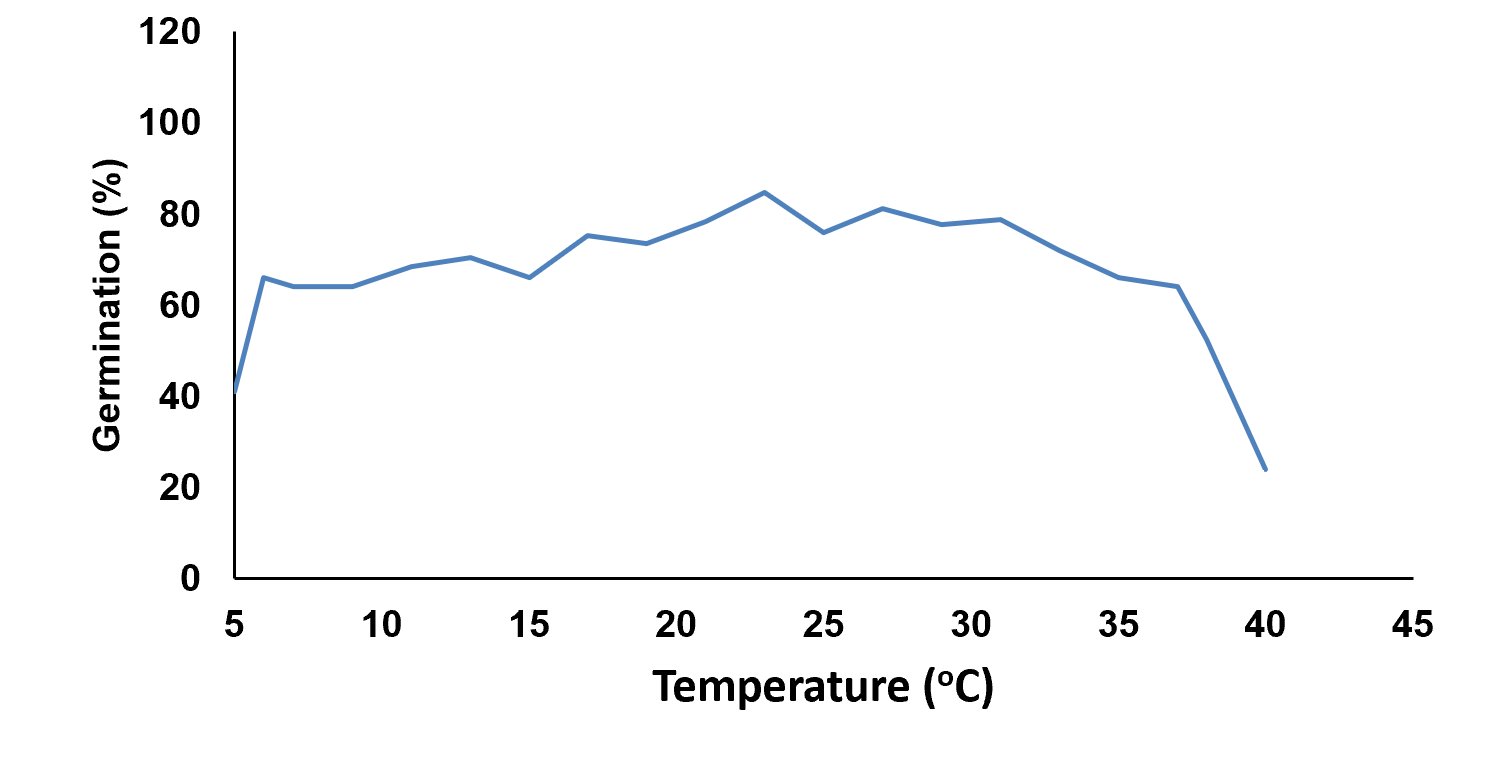

Supplement: Supplementary Figure 1 — Percent germination of Abutilon theophrasti seed at temperatures of 5–40°C. [file Image_1.TIF]

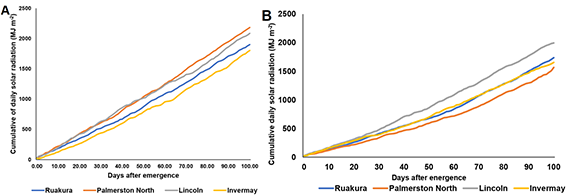

Supplement: Supplementary Figure 2 — The pattern of daily solar radiation (MJ m–2) at the experimental sites in the first (A) and second (B) experiments. [file Image_2.TIF]
