# Supplementary material for: Behaviour of Abutilon theophrasti in Different Climatic Niches: A New Zealand Case Study
Source: Front Plant Sci. 2022 Apr 25;13:885779. doi: 10.3389/fpls.2022.885779 (PMC9083271; doi:10.3389/fpls.2022.885779)
Supplement: Supplementary file 3 [file Table_1.docx]

**Table S1** Average monthly maximum and minimum temperatures at the experimental sites.

|  | Palmerston North | | Ruakura | | Lincoln | | Dunedin | | Woodlands | |
| --- | --- | --- | --- | --- | --- | --- | --- | --- | --- | --- |
|  | Tmax(°C) | Tmin(°C) | Tmax(°C) | Tmin(°C) | Tmax(°C) | Tmin(°C) | Tmax(°C) | Tmin(°C) | Tmax(°C) | Tmin(°C) |
| September-2018 | 15.3 | 6.7 | 18.0 | 6.3 | 14.5 | 4.7 | 13.4 | 5.6 | 14.3 | 0.8 |
| October-2018 | 17.3 | 8.4 | 19.7 | 6.6 | 16.8 | 6.1 | 15.4 | 7.6 | 17.3 | 3.8 |
| November-2018 | 20.0 | 10.7 | 21.6 | 9.6 | 18.3 | 8.6 | 15.9 | 9.5 | 17.0 | 5.7 |
| December-2018 | 22.9 | 13.4 | 24.1 | 13.0 | 19.6 | 12.3 | 18.4 | 11.9 | 21.8 | 9.1 |
| January-2019 | 24.3 | 15.1 | 26.6 | 14.5 | 24.1 | 13.1 | 21.2 | 12.9 | 26.0 | 9.7 |
| February-2019 | 24.3 | 12.5 | 27.5 | 12.7 | 23.9 | 11.6 | 20.2 | 12.1 | 24.1 | 7.5 |
| March-2019 | 23.9 | 12.9 | 25.3 | 13.0 | 22.0 | 11.9 | 20.5 | 12.8 | 23.3 | 7.8 |
|  |  |  |  |  |  |  |  |  |  |  |
| August-2019 | 13.5 | 5.0 | 14.7 | 6.4 | 13.2 | 2.5 | 12.0 | 3.7 | 11.7 | -1.4 |
| September-2019 | 15.0 | 6.8 | 16.5 | 7.2 | 14.9 | 4.7 | 13.4 | 4.7 | 14.3 | -0.1 |
| October-2019 | 16.4 | 8.2 | 17.7 | 9.0 | 15.7 | 6.1 | 13.7 | 6.9 | 15.2 | 2.8 |
| November-2019 | 20.5 | 10.2 | 22.4 | 10.1 | 20.9 | 10.1 | 19.0 | 10.2 | 20.8 | 7.4 |
| December-2019 | 21.3 | 12.5 | 23.4 | 12.5 | 20.8 | 10.3 | 18.1 | 10.1 | 19.3 | 7.2 |
| January-2020 | 22.2 | 13.3 | 24.7 | 11.9 | 22.4 | 11.8 | 18.6 | 12.0 | 25.3 | 9.2 |
| February-2020 | 24.6 | 14.1 | 27.6 | 12.5 | 23.4 | 11.8 | 19.4 | 12.0 | 24.4 | 9.9 |

Tmax = maximum temperature

Tmin = minimum temperature
