# Supplementary material for: Behaviour of Abutilon theophrasti in Different Climatic Niches: A New Zealand Case Study
Source: Front Plant Sci. 2022 Apr 25;13:885779. doi: 10.3389/fpls.2022.885779 (PMC9083271; doi:10.3389/fpls.2022.885779)
Supplement: Supplementary file 4 [file Table_2.docx]

**Table S2** Summary of growing degree days (GDD) values estimated for different trait for the casual and naturalized populations of *Abutilon theophrasti* across five regions in New Zealand in 2018 and 2019.

|  |  | | 2018 | | | | | | | | | | |
| --- | --- | --- | --- | --- | --- | --- | --- | --- | --- | --- | --- | --- | --- |
|  |  | | Casual | | | | |  | Naturalized | | | | |
|  | | Palmerston north | | Ruakura | Lincoln | Invermay | Woodlands |  | Palmerston north | Ruakura | Lincoln | Invermay | Woodlands |
| Onset of emergence | | 88.9 | | 359.4 | 77.1 | 84.9 | 79.5 |  | 88.9 | 359.4 | 89.8 | 84.9 | 79.5 |
| 1^st^ leaf | | 244.2 | | 447.2 | 199.3 | 232.0 | 150.5 |  | 244.2 | 447.2 | 212.9 | 232 | 150.21 |
| 2^nd^ leaf | | 308.1 | | 508.5 | 236.1 | 274.4 | 202.3 |  | 308.1 | 508.5 | 246.9 | 274.4 | 200.4 |
| 3^rd^ leaf | | 368.4 | | 611.1 | 297.6 | 338.7 | 244.2 |  | 368.4 | 611.1 | 304.9 | 335.4 | 246.4 |
| 100 mm height | | 496.7 | | 621.6 | 422.3 | 232.0 | 401.3 |  | 496.7 | 689.8 | 452.5 | 232.0 | 420.7 |
| 200 mm height | | 627.3 | | 846.9 | 521.7 | 274.4 | 531.2 |  | 627.3 | 864.5 | 535.9 | 274.4 | 566.9 |
| 300 mm height | | 713.9 | | 961.9 | 591.2 | 338.7 | 675.5 |  | 760.3 | 961.9 | 632.7 | 335.4 | 735.2 |
| Flowering | | 833.9 | | 961.9 | 564.8 | 953.1 | 823.2 |  | 833.9 | 961.9 | 582.8 | 1063.5 | 852.7 |
| 1^st^ seed capsules | | 1385.7 | | 1417.5 | 1075.9 | 1804.3 | NA |  | 1327.3 | 1432.2 | 1346.4 | 1867.4 | NA |
|  | | 2019 | | | | | | | | | | | |
|  | | Casual | | | | | |  | Naturalized | | | | |
|  | | Palmerston north | | Ruakura | Lincoln | Invermay | Woodlands |  | Palmerston north | Ruakura | Lincoln | Invermay | Woodlands |
| Onset of emergence | | 99.5 | | 106.9 | 94.6 | 39.7 | 28.5 |  | 99.5 | 106.9 | 107.9 | 39.7 | 28.5 |
| 1^st^ leaf | | 293.9 | | 269.9 | 227.6 | 293.9 | 269.9 |  | 321.6 | 275.3 | 238.4 | 321.6 | 275.3 |
| 2^nd^ leaf | | 366.2 | | 576.9 | 295.8 | 366.2 | 576.9 |  | 396.8 | 576.9 | 299.3 | 396.8 | 576.9 |
| 3^rd^ leaf | | 402.3 | | 665.2 | 352.9 | 402.3 | 665.2 |  | 469.3 | 665.2 | 345.2 | 469.3 | 665.2 |
| 100 mm height | | 538.4 | | 617.5 | 487.3 | 645.4 | 538.4 |  | 743.9 | 684.9 | 497.8 | 669.5 | 743.9 |
| 200 mm height | | 765.9 | | 811.4 | 624.7 | 742.7 | 765.9 |  | 879.8 | 865.2 | 655.4 | 797.3 | 879.8 |
| 300 mm height | | 896.7 | | 904.2 | 708.9 | 867.8 | 896.7 |  | 1000.7 | 949.1 | 731.0 | 831.8 | 1000.7 |
| Flowering | | 1033.3 | | 1122.6 | 752.4 | 1133.4 | NA |  | 1082.4 | 1199.6 | 755.0 | 1124.9 | NA |
| 1^st^ seed capsules | | 1328.4 | | 1351.6 | 1570.6 | 1526.9 | NA |  | 1391.2 | 1351.6 | 1664.3 | 1526.9 | NA |

NA = Not available
