# Supplementary material for: Behaviour of Abutilon theophrasti in Different Climatic Niches: A New Zealand Case Study
Source: Front Plant Sci. 2022 Apr 25;13:885779. doi: 10.3389/fpls.2022.885779 (PMC9083271; doi:10.3389/fpls.2022.885779)
Supplement: Supplementary file 6 [file Table_4.docx]

**Table S4 The dates when phonotypic data were collected from five locations for the causal population of velvetleaf.**

| **Location** | **Ruakura** | | **Palmerston North** | | **Lincoln** | | | **Invermay** | | **Woodlands** | |
| --- | --- | --- | --- | --- | --- | --- | --- | --- | --- | --- | --- |
| **Year** | **2018/19** | **2019/20** | **2018/19** | **2019/20** | | **2018/19** | **2019/20** | **2018/19** | **2019/20** | **2018/19** | **2019/20** |
| 100 mm high | 7.11.18 | 21.10.19 | 9.11.18 | 18.10.19 | | 12.11.18 | 11.11.19 | 13.11.18 | 20.11.19 | 4.12.18 | Never |
| 200 mm high | 12.11.18 | 6.11.19 | 21.11.18 | 7.11.19 | | 23.11.18 | 25.11.19 | 3.12.18 | 3.12.18 | 20.12.18 | Never |
| 300 mm high | 21.11.18 | 15.11.19 | 28.11.18 | 22.11.19 | | 29.11.18 | 25.11.19 | 9.12.18 | 9.12.18 | 31.12.18 | Never |
| 1^st^ flower | 26.11.18 | 1.12.19 | 7.12.18 | 6.12.19 | | 27.11.18 | 2.12.19 | 3.1.19 | 6.1.20 | 16.1.19 | Never |
| Height at flowering | 26.11.18 | 1.12.19 | 7.12.18 | 6.12.19 | | 27.11.18 | 2.12.19 | 3.1.19 | 6.1.20 | 16.1.19 | Never |
| Fully developed capsules | 2.1.19 | 19.12.19 | 16.1.19 | 30.12.19 | | 29.1.19 | 5.2.20 | 8.3.19 | 17.2.20 | Never | Never |
| Final height | 2.4.19 | 5.2.20 | 25.3.19 | 24.1.20 | | 15.2.9 | 13.3.20 | 11.2.19 | 24.3.20 | 11.4.19 | Never |
